# *Rubuskaznowskii* (Rosaceae), a new bramble species from south-central Poland

**DOI:** 10.3897/phytokeys.185.71193

**Published:** 2021-11-12

**Authors:** Piotr Kosiński, Tomasz Maliński, Marcin Nobis, Magdalena Rojek-Jelonek, Dominik Tomaszewski, Monika Dering, Jerzy Zieliński

**Affiliations:** 1 Institute of Dendrology, Polish Academy of Sciences, Parkowa 5, 62-035 Kórnik, Poland Institute of Dendrology, Polish Academy of Sciences Kórnik Poland; 2 Faculty of Agronomy and Bioengineering, Poznań University of Life Sciences, Wojska Polskiego 28, 60-637 Poznań, Poland Poznań University of Life Sciences Poznań Poland; 3 Faculty of Forestry and Wood Technology, Poznań University of Life Sciences, Wojska Polskiego 28, 60-637 Poznań, Poland Jagiellonian University Krakow Poland; 4 Department of Plant Taxonomy, Phytogeography & Paleobotany, Jagiellonian University, Gronostajowa 3, 30-387, Kraków, Poland University of Silesia in Katowice Katowice Poland; 5 Institute of Biology, Biotechnology, and Environmental Sciences, University of Silesia in Katowice, Jagiellońska 28, 40-032 Katowice, Poland Institute of Dendrology, Polish Academy of Sciences Poznan Poland

**Keywords:** distribution, ecology, genome size, morphology, taxonomy

## Abstract

Based on field research in south-central Poland, supplemented with a review of herbarium materials, we identified a stable bramble biotype with a range large enough (190 km distance between the outermost stands) to be described as a new regional agamic species, *Rubuskaznowskii***sp.nov.** It belongs to the series Subthyrsoidei(sect. Corylifolii). Although *R.kaznowskii* has a unique combination of features, it can be potentially mistaken for *R.gothicus*. It differs from the latter species in many aspects, including: pruinose primocanes, denser indumentum of the abaxial leaf surface, and more curved prickles on the petiole. *R.kaznowskii* has mainly been observed on rusty soils, in habitats of mixed coniferous and mixed broadleaf forests, usually in sunny places, along forest margins and roads, in clearings and roadside thickets.

## Introduction

The genus *Rubus* is one of the taxonomically most complex group of angiosperms. It encompasses 400–500 sexual species and more than twice as many agamic species (Weber 1995). In Europe, among more than 750 bramble species (Kurtto et al. 2010), there are only four sexual diploids; the remaining species are apomictic polyploids (Kollmann et al. 2000; Potter et al. 2007; Krahulcová et al. 2013; Sochor et al. 2015, 2019). More or less facultative apomixis in the genus enables the emergence of new clonal lineages. The identification of brambles is hindered by their high morphological plasticity, which depends on local environmental conditions and the phenological or developmental phase. Attempts to apply traditional species concept to countless morphological forms of brambles caused long-term stagnation in their systematics, which was broken by the so-called “Weberian reform”. Although every stabilised biotype with a well-defined range deserves a status of agamic species (Haveman and de Ronde 2013), only biotypes with a range exceeding (20–)50 km between the outermost stand are officially described as new taxa (Weber 1995; Holub 1997).

A recent study on *Rubus* flora of Poland is based on this modern concept of agamic species (Zieliński 2004). However, it remains incomplete because not all regions of the country have been thoroughly explored in this respect. Zieliński (2004) mentioned 90 *Rubus* species from Poland. Since this time, 17 other species have been added to this list. Among them, there are 7 newly described bramble species (Zieliński and Trávníček 2004; Zieliński et al. 2004a; Trávníček et al. 2005; Maliński et al. 2014; Wolanin et al. 2016, 2020; Kosiński et al. 2018) and 10 species new for the Polish flora (Zieliński and Trávníček 2004; Zieliński et al. 2004a; 2004b, Trávníček and Zázvorka 2005; Kosiński 2006, 2010; Kosiński and Oklejewicz 2006; Kosiński and Zieliński 2013; Oklejewicz et al. 2013; Kosiński et al. 2014; Maliński et al. 2015). Together with the new taxon described here, the current Polish *Rubus* flora consists of 108 species.

During field studies in the Małopolska Upland (south-central Poland), we came across a remarkable bramble morphotype from the section Corylifolii Lindley, which was different from any other species of the section. The subsequent herbarium survey showed that this bramble had been relatively frequently collected in some areas where detailed floristic investigations were carried out. This allows us to suppose that the so far known area of its occurrence may be still somewhat underestimated.

We classified this bramble to ser. Subthyrsoidei (Focke) Focke (sect. Corylifolii). The series includes species that are supposed to originate by hybridisation of *Rubuscaesius* L. and species of the series *Discolores* (P.J. Müller) Focke or *Rhamnifolii* (Bab.) Focke (Weber 1995; Zieliński 2004). Most of the 37 species known in ser. Subthyrsoidei have their centre of distribution in north-central Europe, mainly Germany, especially the eastern part, Denmark, southern Sweden, south-western Poland, Czechia and Slovakia (see the map “AFE 4586” in Kurtto et al. 2010). The series is represented in Poland by six species; they occur in the western part of the country, particularly in Lower Silesia. Only one species, *R.kuleszae* Ziel., has been confirmed so far in the area covered by the field research under this study (Zieliński 2004). The representatives of the series are characterised by the low-arching first-year stems with relatively uniform prickles and scattered to numerous stalked glands; the terminal leaflets of the first-year stem are typically elliptic or obovate, the leaves are green to grey-tomentose beneath, and long-stalked glands are present on the inflorescence axis and pedicels (Weber 1995; Zieliński 2004).

## Methods

Field studies were conducted mainly during 2014 and 2019. The position of each stand (latitude, longitude and elevation) was determined using a handheld GPS unit. The distribution category was assigned following Weber (1995) and Kurtto et al. (2010). Voucher specimens collected during the studies (including specimens used for morphological investigations) were deposited in the Herbarium of the Institute of Dendrology, Kórnik (KOR). Distribution maps were compiled in the QGIS 3.16 software (QGIS Development Team 2020), using grid squares following the principles presented in the Atlas Florae Europaeae, AFE (Lampinen 2001), and the Atlas of Distribution of Vascular Plants in Poland, ATPOL (Zając 1978; Verey 2017). We also searched for previous records of *R.kaznowskii* from the studied area in the herbarium of the Jagiellonian University (KRA). The morphological description was based on the revision of all herbarium specimens; some characteristics (e.g., features of flowers) were studied on plants growing in the field and in garden collections. We examined well-developed first-year stems (primocanes) and inflorescences. Additional reference material for the comparative study of similar species was obtained from the Herbarium of the Institute of Dendrology in Kórnik (KOR), which has the most comprehensive collection of brambles occurring in Poland and neighbouring areas (accessible at: https://rcin.org.pl/dlibra/collectiondescription/478).

For the nuclear DNA content estimation in *R.kaznowskii*, flow cytometry was used. Leaves of *R.kaznowskii* and the internal standard *Solanumlycopersicum* L. ‘Stupicke’ (1.96 pg/2C DNA; Doležel et al. 1992) were chopped together with a razor blade in a Petri dish in 500 μl of a staining buffer (CyStain PI OxProtect 05–5027). The nuclei suspension was filtered through a 30-μm mesh (CellTrics, Sysmex) into a clean tube. Subsequently, the samples were stained with 1.5 ml of a staining buffer containing propidium iodide and RNase (CyStain PI OxProtect 05–5027) and 1% β-mercaptoethanol (Sigma) and incubated for 50 min at room temperature in the dark. After incubation, the samples were analysed using a CyFlow Space flow cytometer (Sysmex) equipped with a 532-nm green laser. At least 5,000 nuclei were read for each sample; DNA ploidy was established by comparison of the 2C DNA content in the new species with that of the triploid *R.crispomarginatus* Holub, tetraploid *R.prissanicus* Kosiński, Maliński & Ziel. and hexaploid *R.capitulatus* Utsch (Kosiński et al. 2018).

## Taxonomic treatment

### Rubus kaznowskii

Taxon classificationPlantaeRosalesRosaceae

Kosiński & Ziel. sp. nov.

28D7246C-4847-5A05-B0C0-F7EDC4BF636C

urn:lsid:ipni.org:names:77222480-1

Figs 1 , 2 , 3 , 4 , 5 ; Suppl. material 1: Figs S2-S5

#### Type.

**Poland. Łódź Province**: Łask District, 250 m SE of Gucin village, alongside the road in the pine forest, 186 m alt., 51°32'46"N, 19°14'54"E, 14 Jul 2014, P. Kosiński & J. Zieliński s.n.(holotype: KOR 51366; isotype: KOR 55451).

#### Diagnosis.

*Rubuskaznowskii* can be mistaken for the similar *R.gothicus*. However, it differs from the latter species in several aspects. Primocanes of *R.kaznowskii* are pruinose and covered with usually smaller and slightly curved (not straight) prickles, more numerous hairs and stalked glands. Prickles on the petiole are strongly curved and smaller. Similarly, the inflorescence axis in *R.kaznowskii* is covered by smaller prickles and usually more numerous stalked glands than that in *R.gothicus*. The serration of leaf blade margins is more regular and finer; the apex of the terminal leaflet is more gradually narrowed and shorter. The abaxial surface of the lamina (yellowish when dry) is covered by a denser indumentum and has distinct whitish, protruding veins. Although indumentum in both species consists predominantly of fasciculate hairs, their proportion to simple hairs in *R.kaznowskii* is higher than that in *R.gothicus*. The lack of long hairs in the indumentum, both fasciculate and simple, allows distinguishing *R.kaznowskii* not only from *R.gothicus* but also from other Polish representatives of *Subthyrsoidei*. For detailed differences between *R.kaznowskii* and *R.gothicus*, see Table 1.

Some similarities may link *R.kaznowskii* with two other species from the series *Subthyrsoidei*, *R.holandrei* P.J.Müller (= *R.grossus* H.E.Weber) and *R.storhii* H.E.Weber & M.Ranft., which has not been found in Poland until now. Both species differ from *R.kaznowskii* by the absence of stalked glands on the vegetative stems (*R.kaznowskii* – usually numerous), less hairy leaf undersides, straight or slighty curved prickles on the petioles (*R.kaznowskii* – hooked), and flower colours: the former has white, and the latter pink petals (*R.kaznowskii* – white or pinkish-white and pinkish in the bud).

#### Description.

Shrub, usually up to 70 cm tall. First-year stems low arching, 4–6(–8) mm in diameter, angulate, with flat sides (sometimes slightly furrowed or bluntly angled), flushed violet-red or purple on the side exposed to the sun, more or less pruinose, without or with scattered 0.4–0.6-mm long fasciculate hairs (up to 15 per 1 cm length of stem side); stalked glands and acicles up to 0.4 mm long, rather numerous: usually 5–15(–25) per 1 cm length of stem side; prickles somewhat uneven and shorter than stem diameter, (2.5–)3–5(–6) mm long, quite numerous, 10–15(–19) per 5 cm of stem length, usually little curved, rarely straight and declining, abruptly tapering from the 2.5–3.5-mm broad base; intermixed with sparse to quite numerous stout small prickles. Leaves on the first-year stem moderately large (14–)18–20(–25)-cm long, 5-foliolate, indistinctly pedate, with flat or somewhat convex (when alive) leaflets arrangement; leaflets partly imbricate with flat or slightly convex/concave laminas; matt green and subglabrous above (with 0–10 adpressed simple hairs per 1 cm^2^, especially towards leaf margin and on main veins, and with scattered subsessile glands); light or whitish- or yellowish-green and soft to the touch beneath because of dense indumentum (50–90% of the intercostal area) of fasciculate hairs and less numerous simple hairs, with clearly visible bright and protruding veins. Terminal leaflet lamina (7–)8–10(–11)-cm long, usually ovate and widest below the middle (37–50% of its length); shallowly cordate at the base, gradually narrowed, with an apex 8–15 mm long; petiolule moderately, (17–)20–35(–39) mm, long (25–35% of its lamina length). Basal leaflets mostly sessile or sometimes with short petiolules up to 1–2 mm long; their lamina narrowly ovate to obovate, usually shorter than petioles (about 90% of their length on average). Leaf margin periodically serrate, with incisions 2–3(–5) mm deep, sometimes with 1–2 quite distinct lobes near the middle of the lamina; teeth usually triangular, broader than long, with a thin narrow apex. Petioles sparsely hairy, with scattered stalked glands and usually with 9–13 strongly curved, 1–2-mm long prickles. Stipules filiform, with scattered hairs and stalked glands. Inflorescence up to 20–30 cm long, broadly paniculate, rounded near apex, with erectopatent lateral branches up to 7–10 cm long; usually leafless above, with 3(–5)-foliolate leaves below. Inflorescence axis hairy with adpressed small fasciculate (stellate) hairs and scattered longer both fasciculate and simple hairs; stalked glands 50–80 per 1 cm of the axis length, 0.2–0.5 mm long; prickles usually 5–10 per 5 cm of axis length, declining, slender, subulate, slightly curved, 2–3 mm long (1–2 × as long as the axis thickness). Flower pedicels (5–)10–20(–25) mm long, densely hairy and with 40–80 stalked glands; prickles 4–8(–14), slender, slightly curved and declining, 1–2 mm long. Sepals patent after anthesis, 6–9 mm long, grey-green-felted with numerous stalked glands and several pricklets on the outer surface. Petals white or pinkish-white, wrinkled, broadly ovate or obovate, 12–13 mm broad and 14–16 mm long; often present an additional, incomplete whorl composed of smaller petals. Stamens longer or as long as the yellowish-green styles; anthers glabrous, yellowish-white; filaments whitish. Carpels glabrous or sparsely hairy, receptacle usually with long hairs protruding among carpels. Flowering June-July.

**Table 1.** Main morphological differences between *Rubuskaznowskii* and *Rubusgothicus*.

Features	*R.kaznowskii*	*R.gothicus*
**Primocane**		
Wax layer	More or less pruinose	Unpruinose
Hairs (number per 5 cm of the stem length)	(0–)10–50 fasciculate hairs	0–10 simple hairs
Stalked glands (number per 5 cm of the stem length)	25–75	1–10
Prickles	Usually slightly curved and shorter than stem diameter	Usually straight, the longest as long as the stem diameter or longer
**Leaf**		
Apex of the terminal leaflet	Gradually narrowed, 0.8–1.5 cm	Abruptly narrowed, 1.5–2.0 cm
Blade margin of the terminal leaflet	Usually slightly periodical	Usually distinctly periodical
Prickles on petiole	Strongly curved, not longer than petiole diameter	Slightly curved or straight and declining, longer than petiole diameter
Indumentum cover (abaxial side of the lamina)	Dense (above 50%)	Sparse (below 50%)
Long simple (>0.5 mm), large fasciculate (>0.35 mm), and glandular trichomes (abaxial side of the lamina)	Absent	Numerous
Short (<0.25 mm) and medium (0.25–0.5 mm) simple hairs (abaxial side of the lamina)	Numerous	Rare
**Inflorescence axis**		
Prickles length/rachis diameter ratio	1–2	>2
Prickles form	slightly curved	slightly curved or straight and declining
Stalked glands (number per 5 cm of the axis length)	20–40(–50)	10–25(–50)

#### Genome size.

The nuclear DNA content of *R.kaznowskii* is 2C = 1.43 ± 0.01 pg. Comparison of its PI fluorescence with triploid, tetraploid, and hexaploid *Rubus* species, of which ploidy was previously established by chromosome counting, revealed that *R.kaznowskii* is DNA tetraploid. In the European *Rubus* flora, tetraploids predominate (2n = 28) (Kollmann et al. 2000; Potter et al. 2007; Krahulcová et al. 2013; Sochor et al. 2015, 2019). Tetraploidy also prevails in the ser. Subthyrsoidei. In Poland, there are only two pentaploid species of the series, *R.kuleszae* Ziel. and *R.wahlbergii* Arrh., which are distinguished from their remaining representatives by larger leaves (Zieliński 2004).

**Figure 1.**
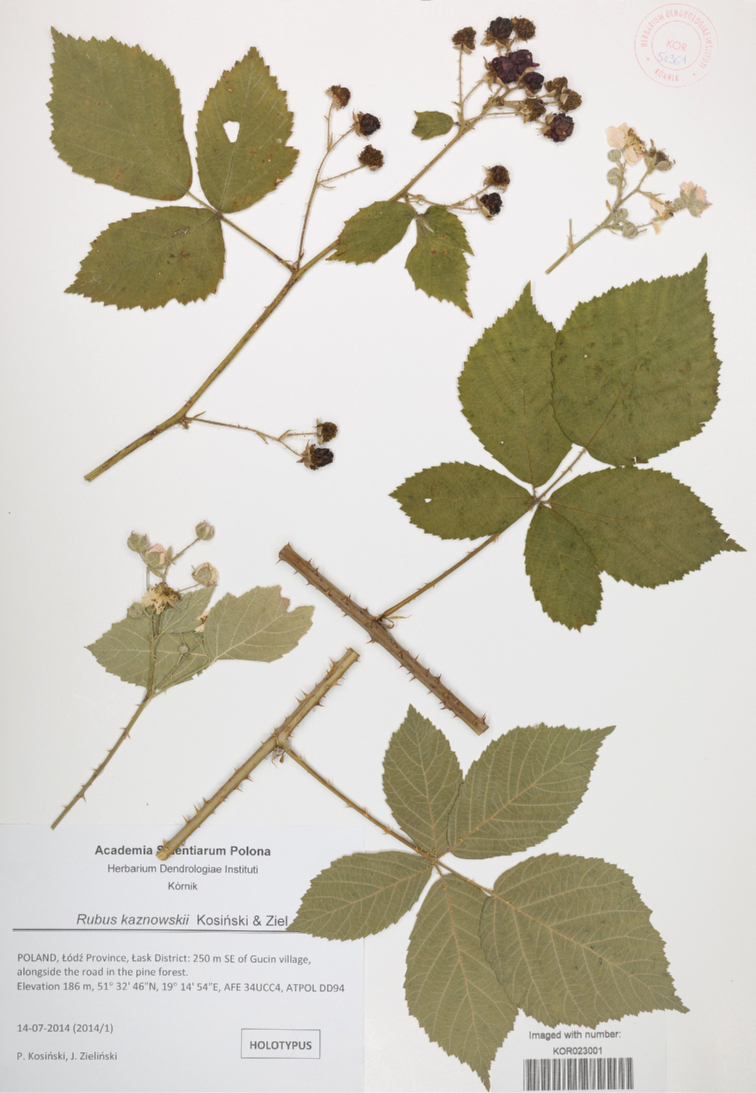
Holotype of *Rubuskaznowskii* (KOR 51366).

**Figure 2.**
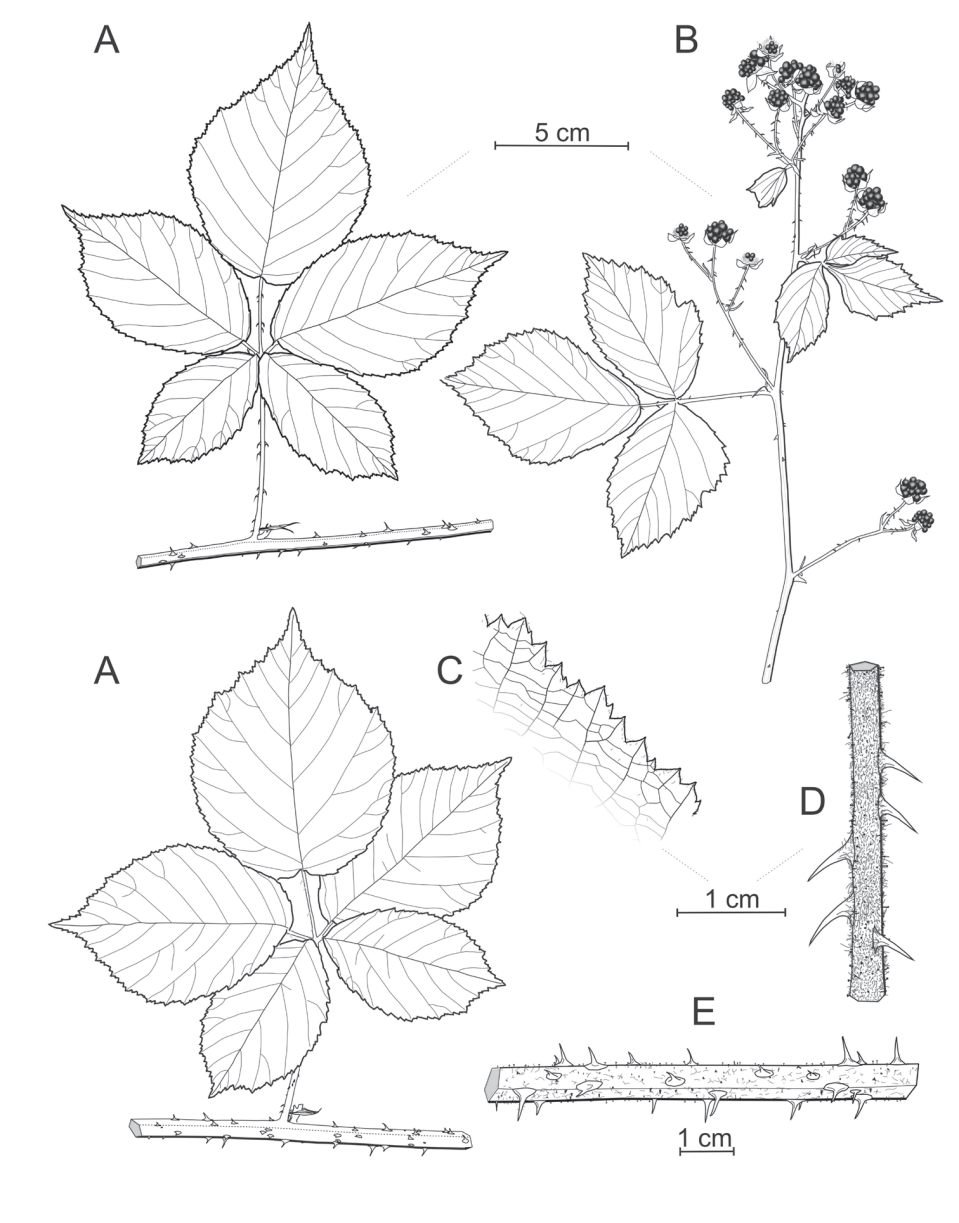
*Rubuskaznowskii***A** segments of the primocane **B** infructescence **C** detail of the terminal leaflet margin **D** detail of the inflorescence axis **E** detail of the first-year stem. Illustration by Piotr Kosiński.

#### Eponymy.

The epithet “kaznowskii” refers to Kazimierz Kaznowski (1876–1943), teacher, naturalist and batologist from the Świętokrzyskie (Holy Cross Mts) region (Poland); the oldest known herbarium specimen of *R.kaznowskii* was collected by him.

**Figure 3.**
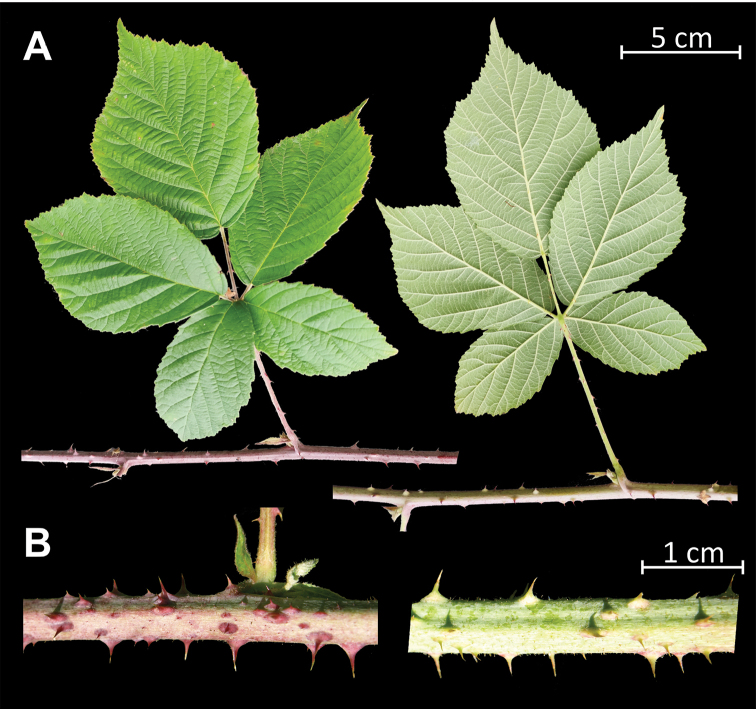
Fragments of primocanes **A** and primocane stems **B** of a living specimen of *Rubuskaznowskii*. Photos by Piotr Kosiński.

#### Distribution and ecology.

*Rubuskaznowskii* is a regionally distributed bramble species. The distance between outermost stands reaches more than 190 km. More than two-thirds of all its stands are located in the Kielce Upland, and one-fifth of them in the Central Masovian Upland, between the Warta and Vistula rivers. It was confirmed in nine AFE units: 34UDB4, 34UEB2, 34UDB1, 34UCC4, 34UDB3, 34UEB1, 34UDB2, 34UEB3, 34UEC2 (Fig. 6). The elevation of *R.kaznowskii* stands ranges from 135 to 325 m. A more detailed map of the distribution of the species in the ATPOL grid is presented in Suppl. material 1: Fig. S1.

**Figure 4.**
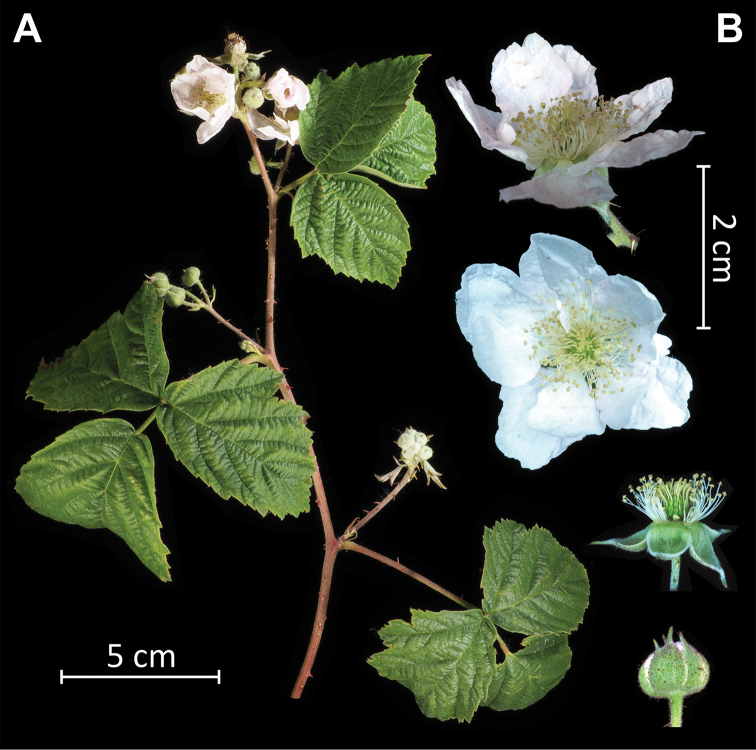
Inflorescence **A** and flowers **B** of a living specimen of *Rubuskaznowskii*. Photos by Piotr Kosiński.

The species occurs mainly on rusty soils (brunic arenosols), in semi-dry to mesic habitats of mixed coniferous and mixed broadleaf forests, preferring open places with favourable light conditions: along forest margins and in clearings and in roadside thickets, among others (Suppl. material 1: Figs S6, S7).

**Figure 5.**
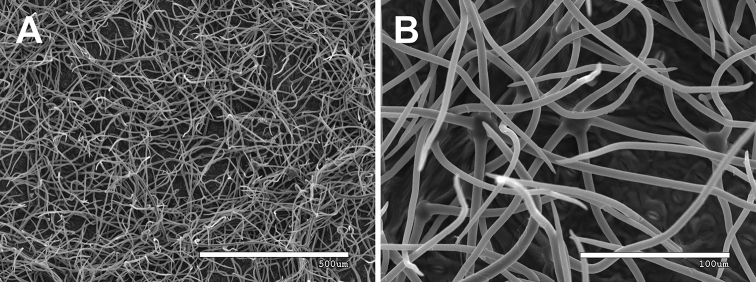
Abaxial side of a leaflet of *Rubuskaznowskii* in SEM. General **A** and more detailed **B** views of indumentum cover in the intercostal area assessed as dense, with the predominance of fasciculate hairs over simple hairs. Photos by Dominik Tomaszewski.

#### Preliminary conservation status.

*Rubuskaznowskii* is a moderately hemerophilic species. No pressures or threats are evidenced. According to IUCN Criteria (IUCN 2021), we propose to include it in the category of Least Concern (LC).

**Figure 6.**
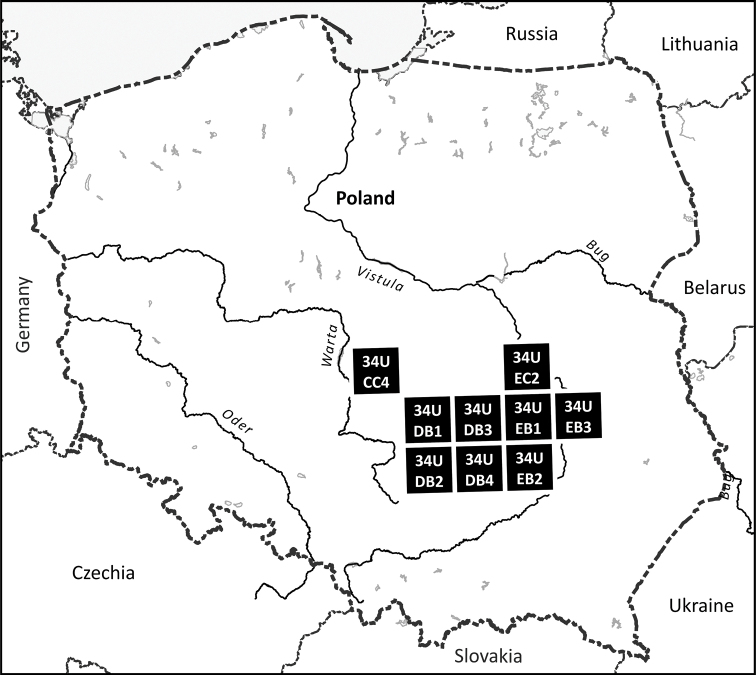
Distribution of *Rubuskaznowskii* based on Atlas Florae Europaeae (AFE) grid system.

#### Specimens examined.

**Poland. Łódź Province**, Łask District: 2 km N of Kamostek, pine-oak forest, roadside thickets, 181 m a.s.l., 51°31'46"N, 19°04'12"E, 10 Sep 2019, *P. Kosiński*, *T. Maliński* & *J. Zieliński* (KOR 55452); Opoczno District: Wierzchowisko, roadside thickets, 228 m a.s.l., 51°13'04.26"N, 20°13'04.45"E, 23 Aug 2010, *A. Trojecka-Brzezińska* (KRA); Piotrków District: 0.5 km NW of Poniatów, broadleaved forest margin, 203 m a.s.l., 51°23'55.33"N, 19°45'02.22"E, 14 Jul 2014, *P. Kosiński* & *J. Zieliński* (KOR 51359); NW outskirts of Poniatów, edge of the brdoadleaved forest, 203 m a.s.l., 51°23'55"N, 19°45'02"E, 14 Jul 2014, *P. Kosiński* & *J. Zieliński* (KOR 51357); SEE of Piotrków Tybunalski, edge of the pine plantation on mixed deciduous forest habitat, 203 m a.s.l., 51°23'46"N, 19°45'29"E, 24 Aug 1999, *J. Zieliński* (KOR 41323); **Mazovia Province**, Białobrzegi District: Ksawerów Stary, roadside thickets, 135 m a.s.l., 51°38'58"N, 21°07'42"E, 13 Sep 2019, *P. Kosiński*, *T. Maliński* & *J. Zieliński* (KOR 55454); Ostrowiec District: Łysowody near Ćmielów, roadside thickets, 203 m a.s.l., 50°54'22"N, 21°33'55"E, 8 Aug 2002, *R. Piwowarczyk* (KRA 0354524); Radom District: W of Młodocin Mniejszy, pine forest, 194 m a.s.l., 51°20'25"N, 21°01'10"E, 3 Aug 2005, *M. Nobis* (KRA); E outskirts of Waliny, alongside the railway and road with the ditch, 189 m a.s.l., 51°21'07.8"N, 21°00'37"E, 4 Aug 2005, *M. Nobis* (KRA); 2.8 km SSE of Maliszów, mixed coniferous forest, 194 m a.s.l., 51°16'08"N, 21°07'53"E, 30 Aug 2002, *M. Nobis* (KRA); Kolonia Dąbrówka Zabłotnia, pine forest margin, 182 m a.s.l., 51°19'06.52"N, 21°03'17.71"E, 24 Jun 2003, *M. Nobis* (KRA 0318820); Kresy near Wierzbica, railway gorge, 203 m a.s.l., 51°16'22.55"N, 21°01'21.32"E, 23 Jun 2003, *M. Nobis* (KRA 0318832); 2 km W of Pakosław, pine-oak forest, 220 m a.s.l., 51°12'22.23"N, 21°07'58.29"E, 2 Jul 2004, *M. Nobis* (KRA 0317627); S of Malczew, sandpit, 179 m a.s.l., 51°21'23.68"N, 21°09'55.12"E, 27 Aug 2005, *M. Nobis* (KRA 0365787); Skarżysko District: N of Kierz Niedźwiedzi, forest margin, 228 m a.s.l., 51°10'53"N, 20°57'06.6"E, 10 Aug 2003, *M. Nobis* (KRA 0318815); Szydłowiec District: Szydłowiec, roadside thickets near the artificial lake, 219 m a.s.l., 51°13'07.16"N, 20°51'20.65"E, 22 Jul 2003, *M. Nobis* (KRA 0320963); 1 km N of Zaława, roadside thickets, 205 m a.s.l., 51°16'04.64"N, 20°45'59.97"E, 22 Jun 2004, *M. Nobis* (KRA 0317573 & 0317574); between Aleksandrów and Budki I, roadside thickets/forest margin, 242 m a.s.l., 51°12'50.98"N, 20°46'18.60"E, 25 Aug 2003, *M. Nobis* (KRA 0318819); Wymysłów, roadside thickets, 218 m a.s.l., 51°14'09.39"N, 20°49'08.00"E, 1 Aug 2003, *M. Nobis* (KRA 0318822); Wola Zagrodnia, roadside thickets, 226 m a.s.l., 51°14'43.59"N, 20°44'49.88"E, 11 Jun 2003, *M. Nobis* (KRA 0321855); Lipienice, alongside railway and road, 205 m a.s.l., 51°14'45.8"N, 20°58'41.6"E, 18 Aug 2002, *M. Nobis* (KRA); W part of Gąsawy Rządowe Niwy, forest margin, 240 m a.s.l., 51°11'57.64"N, 20°55'29.66"E, 31 Jul 2003, *M. Nobis* (KRA 0320129); Gąsawy Rządowe, forest margin, 237 m a.s.l., 51°12'16.47"N, 20°56'46.8"E, 8 Aug 2003, *M. Nobis* (KRA 0318823); Zwoleń District: 0.5 km NWW of Górki, pine plantation on the mixed deciduous forest habitat, 161 m a.s.l., 51°19'59"N, 21°35'39"E, 13 Sep 2019, *P. Kosiński*, *T. Maliński* & *J. Zieliński* (KOR 55453); Przyłęk, alongside the forest road with ditch, 143 m a.s.l., 51°18'30"N, 21°44'50"E, 1 Aug 2012, ? (KOR 55461); **Świętokrzyskie Province**, Jędrzejów District: 3 km NW of Zagórze, roadside thickets, 259 m a.s.l., 50°36'41.4"N, 20°10'33.7"E, 8 Aug 2011, *B. Piwowarski* (KRA); 2 km E of Małogoszcz, 1 km N of Bocheniec, pine-oak forest, 249 m a.s.l., 50°48'37"N, 20°18'27"E, 11 Jun 2011, *G. Łazarski* (KRA 0472546); Kielce District: 5.8 km NNW of Mniów, 300 m of Gliniany Las, silver fir forest/hornbeam-oak forest, 325 m a.s.l., 51°01'50"N, 20°24'28"E, 23 Aug 2012, *M. Podgórska* (KOR 55457); 0.5 km NNE of Milechowy, pine-oak forest, 262 m a.s.l., 50°49'38"N, 20°20'10"E, 4 Sep 2014, *G. Łazarski* (KRA 0472934); between Szewce and Łaziska, forest margin, 306 m a.s.l., 50°51'00.23"N, 20°27'41.69"E, 15 Jul 2014, *P. Kosiński* & *J. Zieliński* (KOR 51383); 2 km SW of Łaziska, Piekoszów, thickets at the edge of the wet, mixed forest, 313 m a.s.l., 50°51'29"N, 20°20'10"E, 2 Aug 2012, *G. Łazarski* (KRA 0472550); W of Niestachów, slopes of Mt Otrocz, pine plantation on the mixed deciduous forest habitat, 321 m a.s.l., 50°50'24"N, 20°42'60"E, 28 Jun 1934, *K. Kaznowski* (KOR 10598); between Raków and Sadków, edge of the pine plantation on the mixed deciduous forest habitat, 276 m a.s.l., 50°42'40.07"N, 21°03'33.83"E, 17 Jul 2014, *P. Kosiński* & *J. Zieliński* (KOR 51365); Masłów, pine plantation on the oak forest habitat, 328 m a.s.l., 50°54'30"N, 20°42'30"E, 12 Aug 1987, *J. Zieliński* (KOR 31452); Stara Słupia, roadside thickets, 319 m a.s.l., 50°51'37.7"N, 21°04'35.5"E, 16 Sep 1986, *R. Kapuściński* (SKPN); Słowik, roadside thickets, 244 m a.s.l., 50°50'08"N, 20°32'24"E, 5 Jul 1932, *K. Kaznowski* (KOR 10609); Końskie District: 2 km NE of Gaworczów, wilderness “Kopaczka”, thermophilous oak forest, 264 m a.s.l., 51°17'34"N, 20°27'34"E, 20 Jul 2012, *M. Podgórska* (KOR 55458); 1 km W of Cisownik, clearing of pine and hornbeam-oak forests, 290 m a.s.l., 51°05'17"N, 20°25'07"E, 18 Jul 2010, *M. Podgórska* (KRA); Opatów District: 2.2 km S of Julianów, edge of the pine-oak forest on the mixed deciduous forest habitat, 194 m a.s.l., 50°52'50"N, 21°36'50"E, 13 Sep 2019, *P. Kosiński*, *T. Maliński* & *J. Zieliński* (KOR 55457); Ostrowiec District: Łysowody near Ćmielów, roadside thickets, 215 m a.s.l., 50°54'07"N, 20°26'32"E, 8 Aug 2002, *R. Piwowarczyk* (KRA 0354532); Łysowody near Ćmielów, roadside thickets, 215 m a.s.l., 50°54'20"N, 21°33'20"E, 8 Aug 2002, *R. Piwowarczyk* (KRA 0354531); Skarżysko District: 1.5 km S of Grzybowa Góra Mała, roadside thickets/pine forest margin, 237 m a.s.l., 51°7'30.42"N, 20°56'29.80"E, 30 Jun 2003, *M. Nobis* (KRA 0318817); Skarżysko-Kamienna, Piękna street, roadside thickets, 228 m a.s.l., 51°07'12.37"N, 20°54'24.81"E, 21 Aug 2003, *M. Nobis* (KRA 0318828 & 0318829); Starachowice District: Jagodne Małe, forest margin, 227 m a.s.l., 51°08'44.49"N, 20°59'41.41"E, 11 Jul 2003, *M. Nobis* (KRA 0320966 & 0320965); Marcinków, railway embankment, 228 m a.s.l., 51°05'34.88"N, 20°58'54.03"E, 9 Aug 2003, *M. Nobis* (KRA 0320960); Wąchock, thickets by ruins of the manor house, 214 m a.s.l., 51°4'41.9"N, 21°00'55"E, 29 Jul 2002, *M. Nobis* (KRA); W of road between Lubienia and Marcule, along the road in the mesic deciduous forest, 242 m a.s.l., 51°04'23.88"N, 21°11'41.77"E, 7 Aug 2002, *M. Nobis* (KRA 0322795 & 0322796); Szydłowiec District: S of Barak Niwy, roadside thickets/forest margin, 252 m a.s.l., 51°10'49.45"N, 20°51'31.49"E, 26 Jun 2003, *M. Nobis* (KRA 0318818); Gąsawy Rządowe Niwy, roadside thickets/forest margin, 237 m a.s.l., 51°11'25.89"N, 20°56'41.12"E, 11 Jul 2003, *M. Nobis* (KRA 0318824).

## Supplementary Material

XML Treatment for Rubus kaznowskii

## Supplementary materials

Supplementary material 1 Map and photos Data type species data https://binary.pensoft.net/file/609800This dataset is made available under the Open Database License (http://opendatacommons.org/licenses/odbl/1.0/). The Open Database License (ODbL) is a license agreement intended to allow users to freely share, modify, and use this Dataset while maintaining this same freedom for others, provided that the original source and author(s) are credited.Piotr Kosiński, Tomasz Maliński, Marcin Nobis, Magdalena Rojek-Jelonek, Dominik Tomaszewski, Monika Dering, Jerzy Zieliński
