# Supplementary material for: Rubuskaznowskii (Rosaceae), a new bramble species from south-central Poland
Source: PhytoKeys. 2021 Nov 12;185:27–41. doi: 10.3897/phytokeys.185.71193 (PMC8604876; doi:10.3897/phytokeys.185.71193)
Supplement: Supplementary material 1 — Map and photos [file phytokeys-185-027-s001.pdf]

Supplementary Material

Title: *Rubus kaznowskii* (Rosaceae), a new bramble species from south-central Poland

Authors: Piotr Kosiński[1,2], Tomasz Maliński[3], Marcin Nobis[4], Magdalena Rojek-Jelonek[5], Dominik Tomaszewski[2], Monika Dering[2,3], Jerzy Zieliński[2]

[1] Faculty of Agronomy and Bioengineering, University of Life Sciences, Wojska Polskiego 28, 60-637 Poznań, Poland, e-mail: kosinski@up.poznan.pl

[2] Institute of Dendrology, Polish Academy of Sciences, Parkowa 5, 62-035 Kórnik, Poland

[3] Faculty of Forestry, Poznań University of Life Sciences, Wojska Polskiego 28, 60-637 Poznań, Poland

[4] Department of Plant Taxonomy, Phytogeography & Paleobotany, Jagiellonian University, Gronostajowa 3, 30-387, Kraków, Poland

[5] Institute of Biology, Biotechnology, and Environmental Sciences, University of Silesia in Katowice, Jagiellońska 28, 40-032 Katowice, Poland

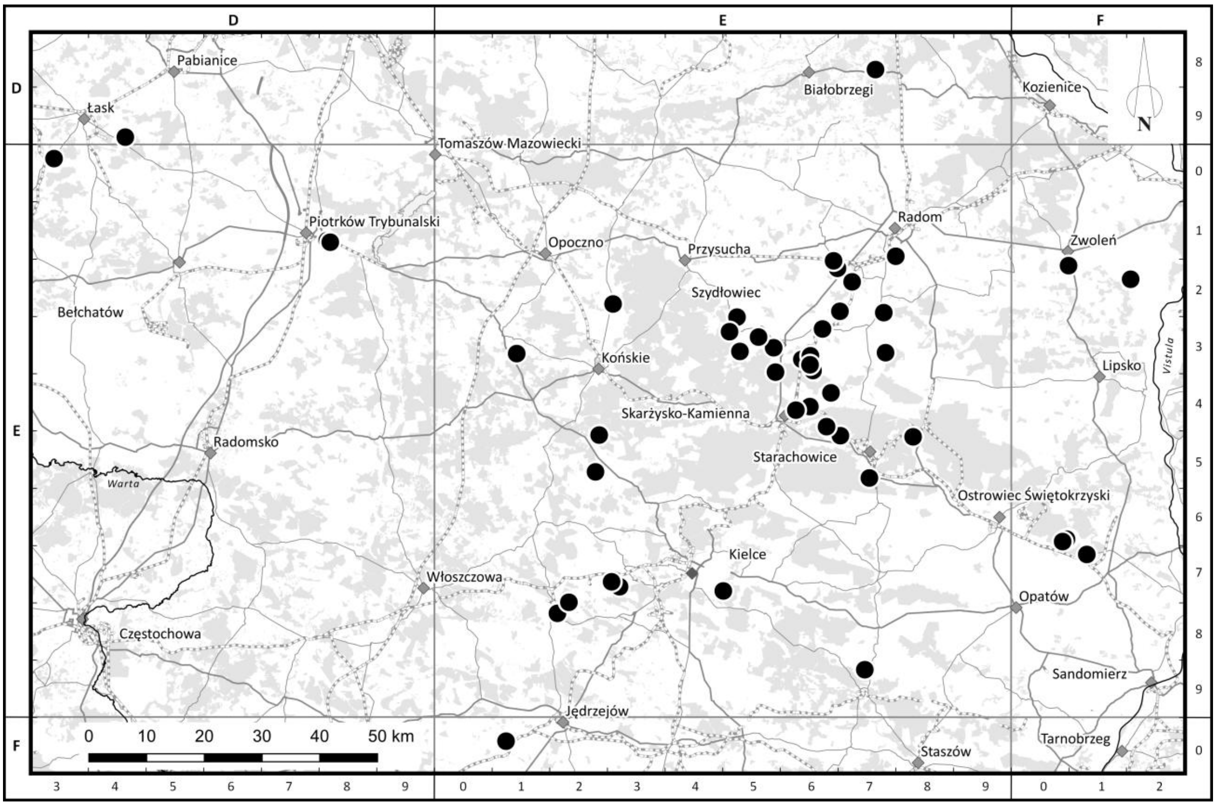

Figure S1. Detailed location of *Rubus kaznowskii* stands (ATPOL grid 10 × 10 km).

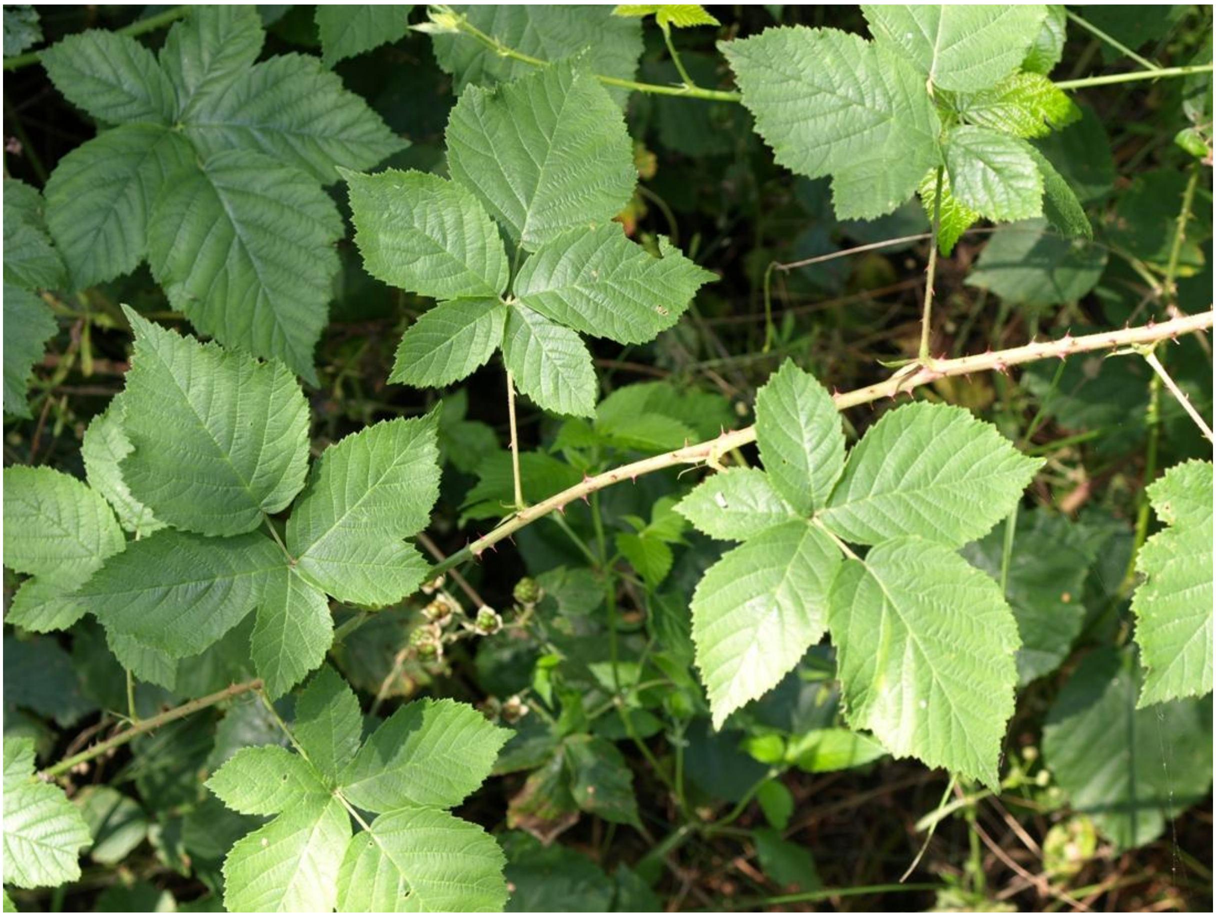

Figure S2. **Primocane of *Rubus kaznowskii* (between Raków and Sadków, 17 Jun 2014).**
Photo by Piotr Kosiński.

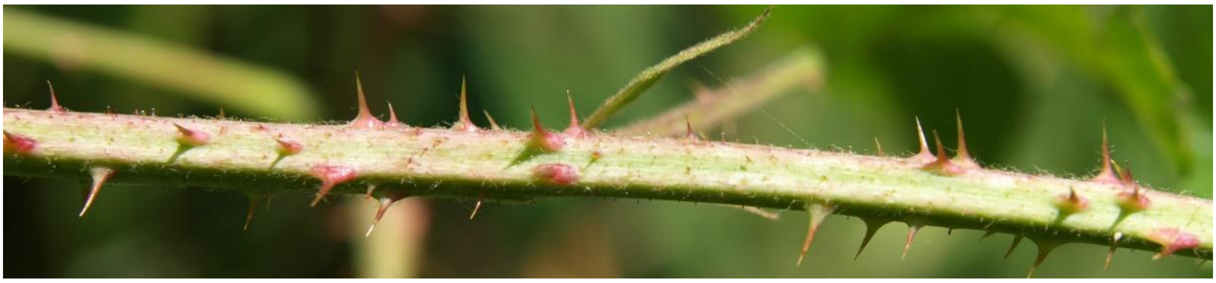

Figure S3. **Young primocane stem of *Rubus kaznowskii* (between Raków and Sadków, 17 Jun 2014).**
Photo by Piotr Kosiński.

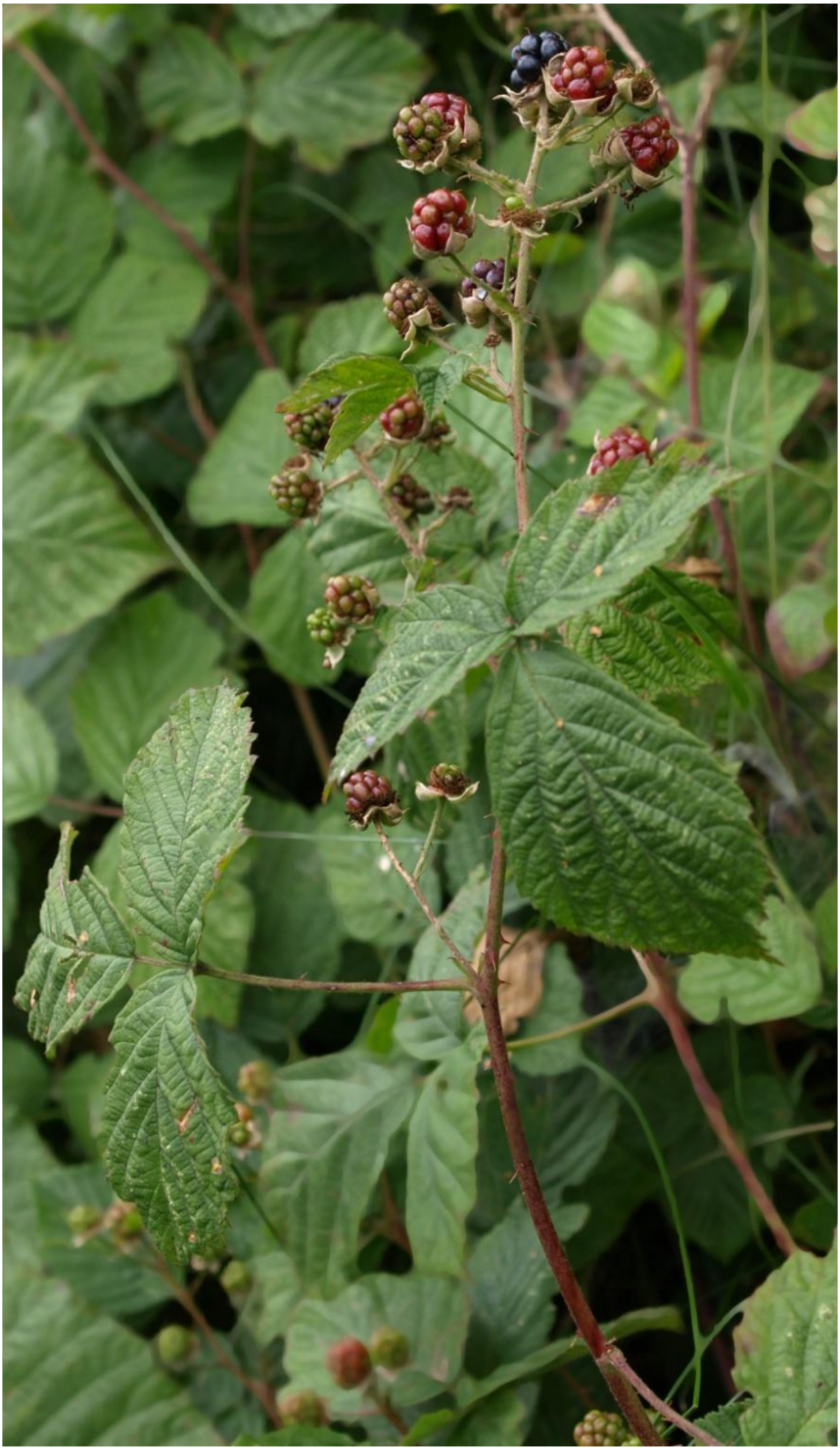

Figure S4. Infructescence of *Rubus kaznowskii* (between Raków and Sadków, 17 Jun 2014). Photo by Piotr Kosiński.

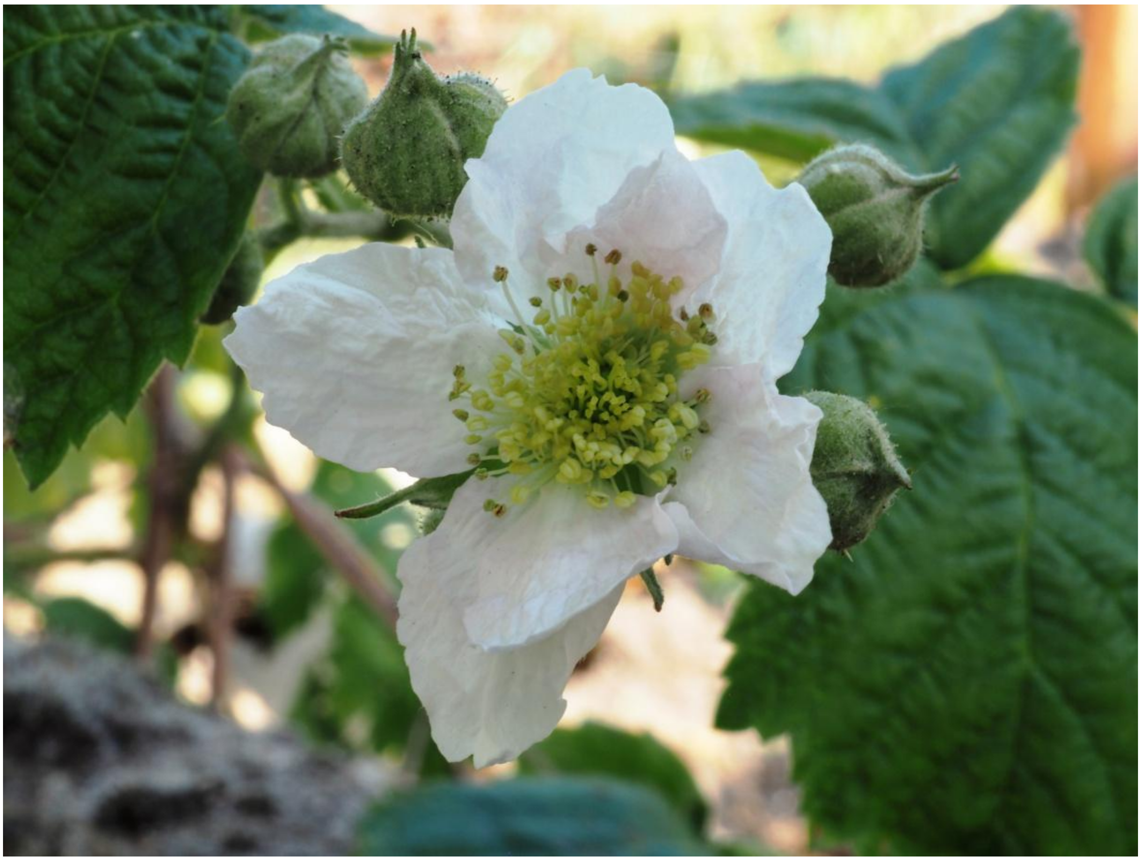

Figure S5. Flower of *Rubus kaznowskii* (garden collection, 11 Jun 2021). Photo by Piotr Kosiński.

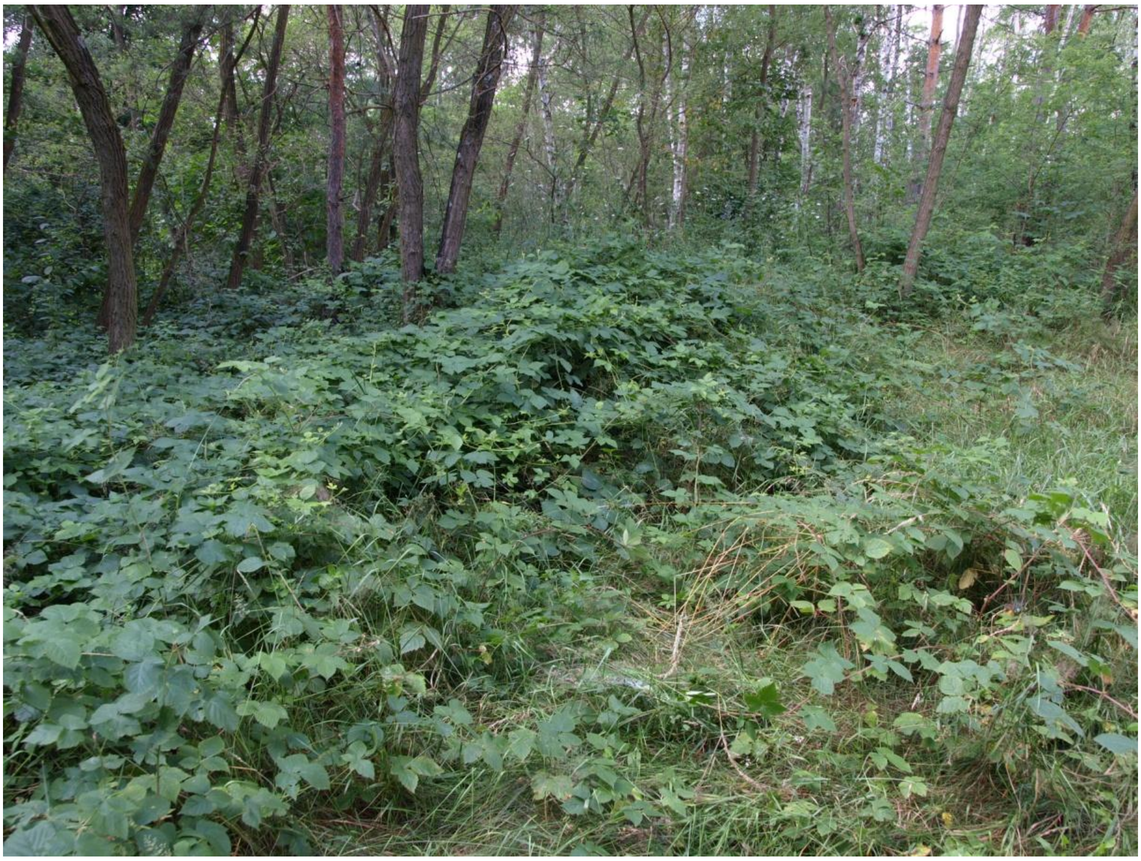

Figure S6. *Rubus kaznowskii* on the edge of the forest (between Raków and Sadków, 17 Jul 2014). Photo by Piotr Kosiński.

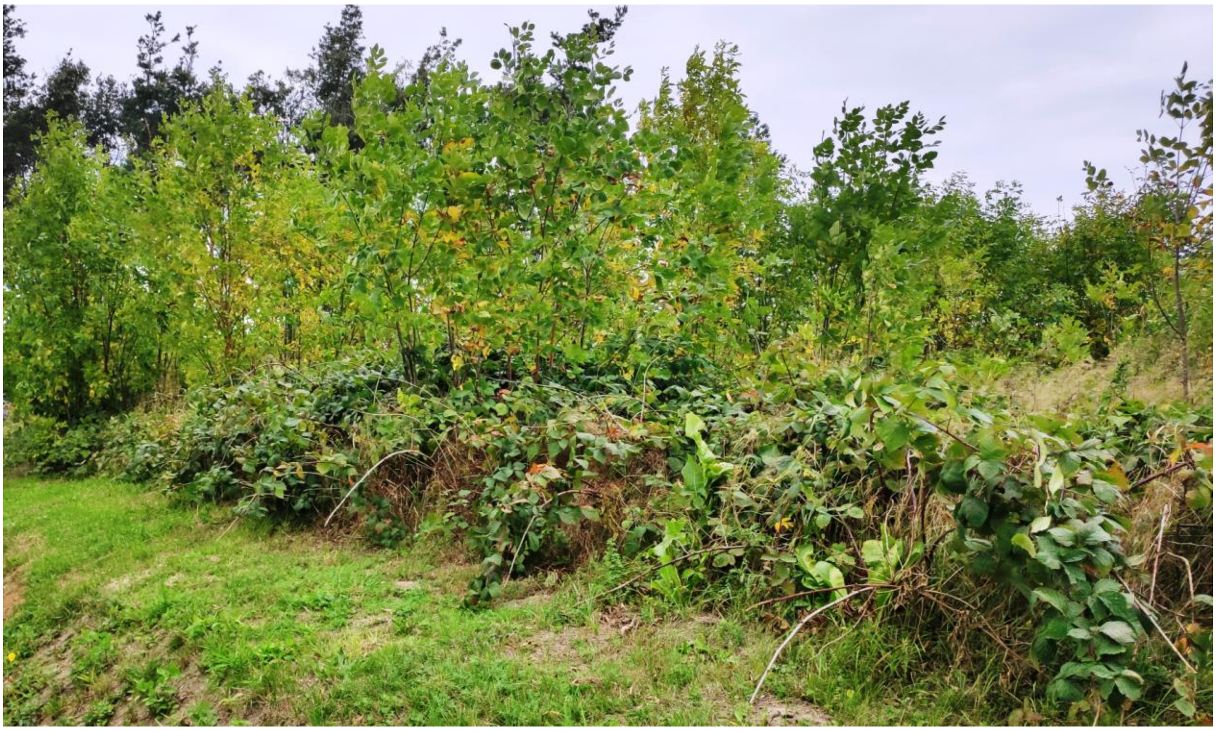

Figure S7. Abundant stand of *Rubus kaznowskii* in a roadside thicket in Ksawerów Stary village (13 Sep 2019). Photo by Piotr Kosiński.
